# A Rare Cause of Acute Kidney Injury in a Female Patient with Breast Cancer Presenting as Renal Colic

**DOI:** 10.1155/2016/9565873

**Published:** 2016-05-17

**Authors:** Roxana Jurubita, Bogdan Obrisca, Gener Ismail

**Affiliations:** Center of Internal Medicine-Nephrology, Fundeni Clinical Institute, 258 Fundeni Street, District 2, 022328 Bucharest, Romania

## Abstract

Renal infarction is a rare cause of acute kidney injury which could lead to permanent loss of renal function. A prompt diagnosis is necessary in order to achieve a successful revascularization of the occluded artery. Given the rarity of the disease and the paucity of the reported cases in the previous literature a high index of suspicion must be maintained not only in the classical cardiac sources of systemic emboli (atrial fibrillation, dilated cardiomyopathy, or endocarditis), but also in the situations when a hypercoagulable state is presumed. The unspecific presenting symptoms often mask the true etiology of the patient's complaints. We present here a rare case of renal infarction that occurred in the setting of a hypercoagulable state, in a female patient with a history of breast cancer and documented hepatic metastases.

## 1. Introduction

Renal infarction is an uncommon cause of acute kidney injury (AKI) and is often misdiagnosed. Although the majority of cases of acute renal artery occlusion occur in the setting of a thromboembolic disease and with blunt abdominal trauma, causing intimal tears and subsequent thrombosis, a small number of cases have been described in hypercoagulable disorders and even in patients with fibromuscular dysplasia [1–3]. The clinical presentation of a patient with renal artery infarction varies, among the most common symptoms being abdominal or flank pain, nausea, vomiting, and gross hematuria. Full recovery of kidney function has been estimated to occur if a revascularization method is undertaken in the first 60 to 90 minutes after a complete artery occlusion; otherwise irreversible renal damage takes place [4]. However, cases of almost full recovery can be seen even when the treatment is delayed by 10–14 days because of a misdiagnosis [5]. This can be explained by the existence of either a collateral artery supplying the kidney or an incomplete occlusion, the cases with total artery occlusion being even rarer than the diagnosis itself. Given the nonspecific presenting symptoms which can mimic nephrolithiasis or pyelonephritis, commonly encountered in clinical practice, and the dependence on an immediate therapeutic intervention in order to avoid prolonged ischemia and loss of renal function, a high index of suspicion must be maintained in patients at risk of a vascular cause of AKI.

Neoplastic disorders have been associated with a high incidence of thromboembolic events during their natural history [6]. The hypercoagulable state that occurs in this context can be attributable to several factors, among those being the malignancy itself (enhanced tissue factor expression by tumor cells), the host's inflammatory response (high levels of tumor cell-derived cytokines, fibrinogen, and von Willebrand factor), and the prothrombotic effects of the treatment [7–9]. Hypercoagulable disorders are commonly associated with venous rather than arterial thrombosis, although cases of renal artery infarction due to heparin-induced thrombocytopenia, antiphospholipid syndrome, or nephrotic syndrome have been described [10, 11]. We describe here a rare case of renal infarction that occurred in the setting of a hypercoagulable state, in a female patient with a history of breast cancer and documented hepatic metastases.

## 2. Case Presentation

A 65-year-old woman presented to the emergency room (ER) with a 6-day history of right flank pain, fever (up to 39°C), nausea, and oliguria. Prior to the admission she was investigated at two other emergency departments, where the biological panel revealed increased serum creatinine (2,9 mg/dL), leukocytosis, and gross hematuria. She was known to have a normal renal function prior to the admission to the first ER. Despite this, she was given a conservative treatment and was discharged home. Her past medical history included breast cancer, for which she underwent surgery, chemotherapy (with exemestane for the previous 4 years), and radiotherapy, congestive heart failure, essential hypertension, and type 2 diabetes mellitus. Her physical examination disclosed an altered general status, normal body temperature, induration of the right breast, pitting edema, blood pressure 140/90 mmHg, and pulse 90/min. The auscultation revealed regular heartbeats with an ejection systolic murmur in the aortic area and diminished breath sounds corresponding to the inferior right hemithorax. The palpation showed minor abdominal tenderness in the right upper quadrant and right flank and hepatomegaly with the lower liver edge at 4 cm below the costal margin. Oliguria and gross hematuria were also noted.

Initial testing showed mild leukocytosis with increased serum creatinine (2,3 mg/dL), urea (186 mg/dL), potassium level (5,8 mmol/L), a severe hyponatremia (122 mmol/L), a serum lactate dehydrogenase of 2900 U/L, and fibrinogen level of 480 mg/dL. The coagulation profile performed showed INR, proteins C and S, and antithrombin III within the normal range. Liver function tests revealed marked cytolysis with ALT 417 U/L, AST 237 U/L, and total bilirubin of 1,9 mg/dL, while the lipid panel displayed total cholesterol of 83 mg/dL and triglycerides of 182 mg/dL. Urinalysis was positive for red blood cells (2078 cells/*μ*L) and leukocytes and protein (albumin/creatinine ration of 200 mg/g). The EKG displayed a normal sinus rhythm with a bundle branch block. The blood and urine cultures were negative.

**Figure 1:**
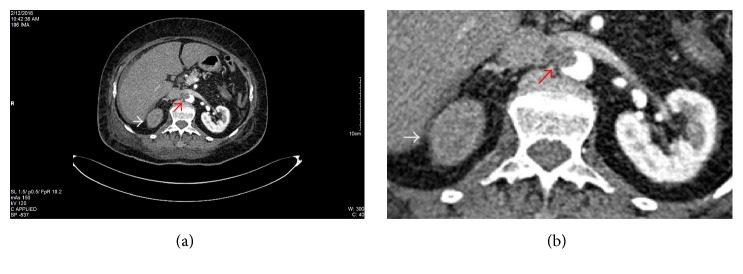
(a) Contrast-enhanced CT scan. Right renal artery occlusion with endoluminal aortic thrombus. The red arrow marks the endoluminal aortic thrombus at the emergence of the right renal artery. The white arrow shows the absence of nephrogram of the right kidney suggestive of renal infarction. (b) An enlarged image of (a) in which the red arrow marks the endoluminal aortic thrombus and the white arrow shows the right kidney with the absence of the nephrogram.

**Figure 2:**
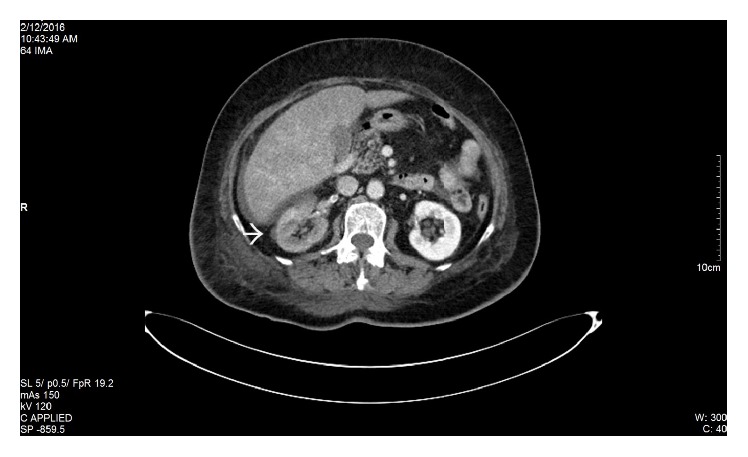
Contrast-enhanced CT scan. Decreased uptake of contrast in the right kidney. The white arrow shows the right kidney with small areas of contrast uptake suggestive of a partial occlusion of the right renal artery.

**Figure 3:**
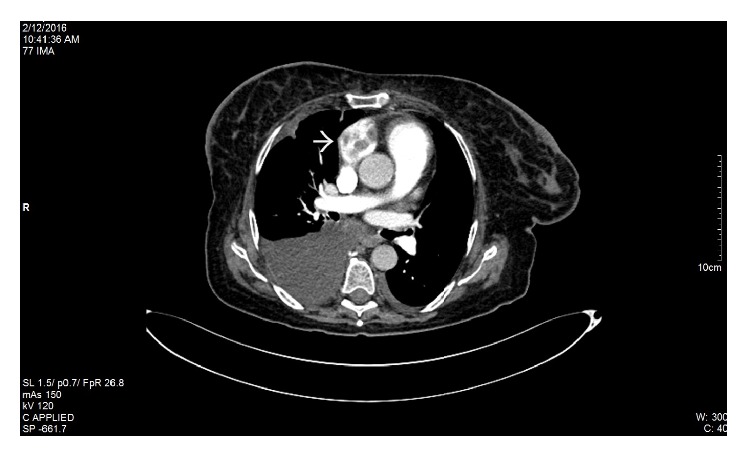
Contrast-enhanced CT scan. The white arrow shows multiple thrombi in the left atrium.

Pyelonephritis and renal colic were at first suspected. Therefore, the initial workup included an abdominal ultrasound, which revealed several hepatic metastases, but did not show any renal abnormalities. Given the patient's past medical history and the clinical presentation, a contrast-enhanced CT scan was needed in order to identify the etiology of the right flank pain. The result showed a decreased uptake in the right kidney suggestive of an acute renal artery occlusion, an endoaortic thrombus localized at the emergence of the right renal artery, and several intracardiac thrombi (Figures 1(a), 1(b), 2, and 3). Additionally, the CT scan identified bone metastases. The echocardiography revealed the same aspect of multiple intracardiac thrombi and moderate calcification of mitral valve leaflets, but without any suggestive images of valvular vegetations.

The diagnosis of acute renal infarction secondary to a cardiac originating embolus was made. The patient was started on low molecular weight heparin for 7 days and then switched to acenocoumarol. A severe vitamin K deficiency was suspected in the setting of protein malnutrition secondary to a metastatic disease. As a consequence, the oral anticoagulants were withdrawn for 5 days and then readjusted for a dose of 1 mg per week. During the hospitalization period, the renal function partially recovered and the serum creatinine stabilized at 1,61 mg/dL and urea at 120 mg/dL. The patient was discharged with the recommendations to continue the anticoagulant treatment and was redirected to an oncology department for specific management. The overall prognosis was poor, given the context of a generalized metastatic breast cancer.

## 3. Discussion

Renal infarction is an unusual cause of acute kidney injury; therefore the diagnosis is frequently delayed. However, its true incidence appears to be higher since a great proportion of cases are discovered incidentally at the autopsy [1]. The main cause of acute renal infarction is reported to be atrial fibrillation [12, 13], with approximately 2 percent of the patients being predisposed to have a renal thromboembolic event during the natural history of the disease [14]. Dilated cardiomyopathy [4, 5, 15], infective endocarditis, or thrombi of atheroma from the suprarenal aorta [1] are other potential sources of emboli to a renal artery. Nevertheless, a retrospective study of 94 patients identified arterial thrombosis secondary to blunt abdominal trauma as being the major cause for renal infarction (30,8%) [1].* In situ* thrombosis of a stenotic renal artery has also been reported in a minority of cases [16] and also in the setting of hypercoagulable states like nephrotic syndrome or heparin-induced thrombocytopenia [7, 10, 11]. Our patient had intracardiac and endoaortic thrombi, despite having a normal sinus rhythm, demonstrating the procoagulant environment maintained by a neoplastic disorder. The metastatic breast cancer, which can lead to a constitutively expressed tissue factor [6, 7], the increased fibrinogen level, and the long-term treatment with exemestane [8, 9] could provide arguments for our hypothesis of a hypercoagulable state. Nevertheless, recent studies have reported that the incidence of thromboembolic events is lower in patients receiving exemestane alone than in those receiving the sequential treatment (tamoxifen for 2-3 years before exemestane) [8, 9]. Nonbacterial thrombotic endocarditis (NBTE) has been classically described in patients with neoplastic diseases [7]. In our case the CT scan and echocardiography could not support the diagnosis of NBTE given the multiple intracardiac thrombi and rather unaffected valve leaflets, leaving the hypercoagulable state in cancer patients as the most likely explanation for the embolic cause of AKI. Although the link between thromboembolic events and malignant disorders is well known, the underlying mechanisms are inadequately understood [6].

Early diagnosis of renal infarction is often difficult to make due to several confounding factors. First, the main clinical presenting symptom is acute onset of abdominal or flank pain, which is frequently misinterpreted as renal colic. In such case, the abdominal ultrasound can be normal and the patient is often prescribed analgesics or anti-inflammatory agents. Fever is also reported in up to 30% of the patients further leading to confusion with pyelonephritis [4]. Gross hematuria often orientates the initial workup but is not usually present in cases of complete arterial occlusion. Anuria or oliguria is suggestive of bilateral kidney involvement or unilateral occlusion in patients with a solitary kidney, although other additional causes of acute renal failure have been described (contrast nephropathy or a reflex arteriolar vasospasm of the contralateral kidney) [17]. Our patient had right arterial thrombosis and transient oliguria. Beside reflex arteriolar vasospasm, our patient had other possible explanations for the deterioration of renal function and oliguria, including a history of diabetic nephropathy, renal hypoperfusion due to the congestive heart failure, and chronic tubulointerstitial nephritis. However, the normal renal function prior to the admission to the first emergency department and moderate proteinuria rule out a severe diabetic nephropathy as a possible coexistent cause of the altered renal function. The most common laboratory abnormality is a marked LDH elevation, a marker of cell necrosis. However, a high level of lactate dehydrogenase in our case can be misleading since this can occur both in a neoplastic disease and in renal infarction. The classical triad of flank/abdominal pain, hematuria, and LDH elevation, although unspecific, should prompt the decision to perform a contrast-enhanced CT scan. Nevertheless, a contrast-enhanced CT scan in a patient with acute kidney injury can further deteriorate the renal function, but, in order to avoid a contrast-induced nephropathy, we have performed a prevention protocol consisting of hydration therapy and administration of N-acetylcysteine. Our patient did not show any deterioration of renal function following the CT scan.

Therapeutic options in acute renal artery occlusion include anticoagulant therapy, intra-arterial thrombolysis, and surgical embolectomy, but, given the rarity of this disorder, a treatment guideline has not been published. The choice between these three approaches depends primarily on the time delay in establishing the diagnosis and the general status of the patient. It is widely accepted that renal parenchyma resists complete ischemia for 60 to 90 minutes; beyond this point irreversible kidney damage occurs [4]. Our patient had in fact an incomplete renal artery occlusion that allowed for a partial recovery of the renal function (a decrease in serum creatinine and restoration of diuresis), in spite of more than 6 days of ischemia. We chose anticoagulation therapy instead of a more invasive approach given the background of the patient and the altered general state, with multiple organ deficiencies. Concerns about the bleeding risk of a patient with metastatic cancer receiving warfarin therapy exist, although several studies did not confirm an increased likelihood of hemorrhagic complications in such patients, excepting the patients with tumor or metastases of the brain [6]. The fact that our patient had only several small hepatic metastases allowed us to choose an oral anticoagulation therapy.

In conclusion, a time-dependent diagnosis is required in order to avoid irreversible loss of renal function in patients with renal infarction. Although thromboembolic diseases related to a cardiac structural or functional defect represent the majority of cases of acute renal artery occlusion, a high index of suspicion must also be maintained in patients with hypercoagulable states at risk of* in situ* thrombosis or embolic events.
